# Novel *GLA* Mutation Promotes Intron Inclusion Leading to Fabry Disease

**DOI:** 10.3389/fgene.2019.00783

**Published:** 2019-09-27

**Authors:** Patrícia Varela, Myrtes Martins Caldas, João Bosco Pesquero

**Affiliations:** ^1^Center for Research and Molecular Diagnostic of Genetic Diseases – Department of Biophysics, Federal University of São Paulo, São Paulo, Brazil; ^2^Department of Nephrology URONEFRO – Hemodiálise e Especialidades, Belém, Brazil

**Keywords:** Fabry disease, *GLA* gene, intron inclusion, splice site mutation, α-galactosidase A

## Abstract

Fabry disease (FD) is a rare and underdiagnosed X-linked disorder resulting from the deficient activity of the lysosomal hydrolase α-galactosidase A, which leads to storage of complex glycosphingolipids inside of lysosomes in critical organs and tissues, impairing their functions and consequently resulting in a progressive multisystem disease. FD is caused by mutations in the *GLA* gene, and only 4.6% of described mutations are located in the splice site regions. RNA splicing is an essential step to the formation of functional proteins, and mutations in splice site regions can cause formation of aberrant transcripts leading to disease. Here we report a novel *GLA* insertion at position c.801+3 in intron 5 (c.801+2_801+3insT) in a Brazilian family with suspicion of FD. The index case, a 46-year-old male, presented undetectable α-galactosidase A activity. Analysis of blood cDNA found two aberrant *GLA* transcripts. In the first transcript, a novel donor splice site was created promoting formation of an intron inclusion with 37 bp. The splice site was not recognized in the second transcript and the intron 5 was not excised. The wild-type transcript was not formed and both aberrant transcripts lead to a premature stop codon. Despite not being in the canonical site, this new mutation disrupts existing 5’ splice site and produces two aberrant transcripts leading to FD.

## Introduction

Fabry disease (FD—OMIM 301500) is a rare X-linked lysosomal storage disease caused by the deficiency or absence of α-galactosidase A enzyme (α-Gal A; EC 3.2.1.22). This defect promotes inability of the lysosomes to catabolize glycosphingolipids, mainly globotriaosylceramide (Gb3), leading to storage of this substrate inside of cells of critical organs and tissues, resulting in a progressive multisystem disease (Auray-Blais et al., 2010; Boutin et al., 2012). FD usually affects males; however, due to X-chromosome inactivation, heterozygous females may exhibit disease manifestations presenting both mild and late symptoms as severe phenotypes as found in males (Wang et al., 2007; Juchniewicz et al., 2018).

The enzyme defect is caused by mutations in *GLA* gene. More than 960 variants were described in the Human Gene Mutation Database (HGMD). Of these, approximately 75% are point mutations, predominantly consisting of missense mutations; nonsense comprises 14% of total mutations, while splicing region mutations comprise only 4.6% of the variants described in the *GLA* gene (Stenson et al., 2009).

It is known that a pre-mRNA splicing, which consists in an excision of the introns with later connection of exons, is crucial for the correct protein production. Mutations in splicing site regions may have effects in the RNA processing such as deletion of part of exons, exon skipping, intron inclusions, and cryptic exon or pseudoexon formation, thus leading to a mature protein that is nonfunctional (Abramowicz and Gos, 2018).

In the *GLA* gene, 48 mutations in splice regions were described; 34 are located in canonical splice regions, at the positions +1 and +2 in the donor splice sites (5’ss) and –1 and –2 in the acceptor splice sites (3’ss). The other 14 mutations, which cause splicing errors, are located in regions close to the canonical sites or in deep intronic regions (Stenson et al., 2009). Although described, many mutations in the splicing site regions in the *GLA* gene were poorly studied.

Here, we present a novel mutation in the intron 5 at position +3 (c.801+2_801+3insT), outside the canonical splicing site, which promotes alteration of the WT donor site affecting splicing of the intron 5. We demonstrate that the sequence close to the canonical site of 5’ss is highly conserved and recognized by the elements of the spliceosome, and therefore, variants in this region may lead to FD.

## Materials and Methods

### Patients

We analyzed a family with suspicion of FD after the identification of predictive clinical symptoms and undetected α-Gal A activity in the index case. Written informed consent was obtained from all individuals included in the study. The Medical Research and Ethical Committee of Universidade Federal de São Paulo approved the research protocols and consent forms used in this study (0585/07 and 0354/18).

### DNA and RNA Isolation

Peripheral blood samples were collected using Ethylenediamine tetraacetic acid (EDTA) as an anticoagulant for DNA isolation and *GLA* sequencing. Genomic DNA was extracted from blood using QIAamp DNA Blood Mini (Qiagen), according to the manufacturer’s conditions. Total RNA was isolated from peripheral blood sample collected in Tempus™ blood RNA tubes (Applied Biosystems) and isolated with Tempus™ Spin RNA Isolation kit (Applied Biosystems), according to the manufacturer’s conditions. The entire coding sequence of *GLA* mRNA was reverse transcribed to produce cDNA.

### Polymerase Chain Reaction (PCR)

The seven exons of *GLA* gene were amplified by polymerase chain reaction (PCR). Forward and reverse oligonucleotide primers were synthesized based on the sequences flanking the seven exons of the *GLA* gene as described by Turaça et al. (2012). The single-stranded cDNA fragments were amplified using specific sense and antisense primers (cDNA 1F-5’ ATG CAG CTG AGG AAC CCA GAA C-3’; cDNA 1R-5’ GTC TGC CTG AAG TCT GCC TT3’; cDNA 2F-5’ GTT GGA TGG CTC CCC AAA GA 3’; cDNA 2R-5’ CAG CCA TGA TAG CCC AGA GG 3’; cDNA 3F-5’ TTG GCC TCA GCT GGA ATC AG 3’, and cDNA 3R-5’ CCC CTC GAG TTA AAG TAA GTC TTT TAA TGA 3’). Exxtend, Paulínia, São Paulo (www.exxtend.com.br). Briefly, the extracted RNA was reverse transcribed to complementary DNAs (cDNA) using M-MLV (Invitrogen, California, EUA) following the manufacturer’s instructions. The cDNA was amplified by PCR at 95°C for 5 min, and 35 cycles (95°C for 30 s, 60°C for 30 s, 72°C for 45 s). After PCR, 20 μl of each sample was subjected to electrophoresis on 1% agarose gel. Amplification products were identified according to the expected molecular weight.

### Sequencing and Bioinformatics Analysis

Each amplicon was purified using QIAquick Gel Extraction kit (Qiagen, Hilden, Germany) according to the manufacturer’s conditions and sequenced using the BigDye Terminator v3.1 cycle sequencing kit and ABI Prism 3500xl Genetic Analyzer sequencer. Data analysis was carried out using software Geneious 10.2.3 (https://www.geneious.com). Sequences were compared with the DNA (NG_007119) and RNA (NM_000169) reference sequence and confirmed by reverse strand sequencing.

Variants were reviewed and annotated using dbSNP [single-nucleotide polymorphism database (ncbi.nlm.nih.gov/projects/SNP/)] and HGMD (Human Genome Mutation Database professional, biobaseinternational.com/product/hgmd). Gnomad (Genome Aggregation Database, gnomad.broadinstitute.org), ABraOM (Brazilian genomic variants, http://abraom.ib.usp.br), and 1000 genomes (http://phase3browser.1000genomes.org) were used to achieve known population frequency. The web-software Human Splicing Finder (Desmet et al., 2009) was used to identify significant splicing motif alterations.

## Results

### Patients and DNA Sequencing

**Figure 1:**
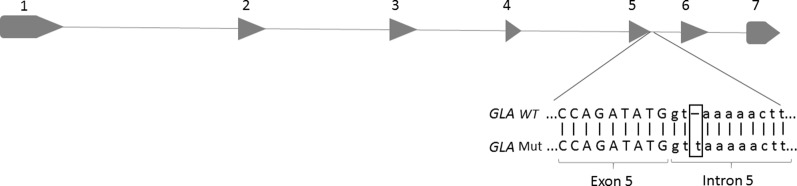
Location of a base insertion (T) in the *GLA* genomic DNA sequence, corresponding to intron 5 of the *GLA* gene (c.801+2_801+3insT). The figure highlights the insertion of a thymine base at the splicing donor site of intron 5.

The index case here evaluated was a 46-year-old male, diagnosed as having FD by clinical signs and undetectable α-Gal A activity. In order to identify the *GLA* mutation underlying to FD in the index case, total DNA was isolated and the sequencing was performed. As seen in **Figure 1**, a base thymine was inserted at the position c.801+3, three nucleotides into the intron 5 after the coding position, located close to the 5’ss. This mutation was not described in the literature and was absent in the three population databases consulted. The Human Splicing Finder predicted this insertion in donor site as most probably affecting the splicing. After finding the mutation in the index patient, the family was also analyzed by DNA sequencing. The mutation was found in the brother and cousin of the index case; both presented FD symptoms. The patients’ clinic is shown in **Table 1**.

**Table 1:** Clinical and laboratory characteristics of individuals with novel *GLA* variant.

	Patient	Index case	Brother	Cousin
**General information**	Gender	Male	Male	Female
	Age	49 years	38 year	49 years
	Onset age of symptoms	21 years	16 year	17 years
	Age at diagnosis	47 years	37 years	48 years
	Enzyme activity (2.2 µmol/h/L)	Undetectable	0.10	NA
**General symptoms**	Facial appearance	–	–	–
	Angiokeratoma	+	+	+
	Oedema	+	–	–
	Musculoskeletal	–	+	–
	Cornea verticillata	–	–	+
	Diaphoresis	–	–	+
	Abdominal pain	–	–	+
	Diarrhea/constipation	+	+	+
	Hemorrhoids	–	–	–
	Pulmonary	–	–	–
	New York Heart	–	–	–
**Neurological symptoms**	Tinnitus	+	–	+
	Vertigo	–	–	–
	Acroparaesthesia	+	+	+
	Fever pain crisis	–	+	–
	Cerebrovascular	+	+	–
	Depression	+	–	–
	Fatigue	+	+	+
	Reduced activity level	+	+	NA
**Renal symptoms**	Microalbuminuria (mg/24 h)	58	56	46
	24 h proteinuria (mg/24 h)	132	129	119
	Creatinine (mg/dl)	1.4	0.6	0.7
	Kidney cysts	–	+	–
	Evidence of renal dysfunction	Increased creatinine levels	Right kidney with scar/sequel appearance	No
**Cardiovascular symptoms**	Changes in cardiac muscle thickness	–	–	–
	Valve Insufficiency	–	–	–
	ECG abnormalities	+	–	–
	Pacemaker	–	–	–
	Hypertension	–	–	–
	**Enzyme Replacement Therapy (ERT)**	Yes	Yes	Yes

Legend: y, years; (+) presence of symptom; (–) absence of symptom; NA, not applicable.

### RNA Analysis

**Figure 2:**
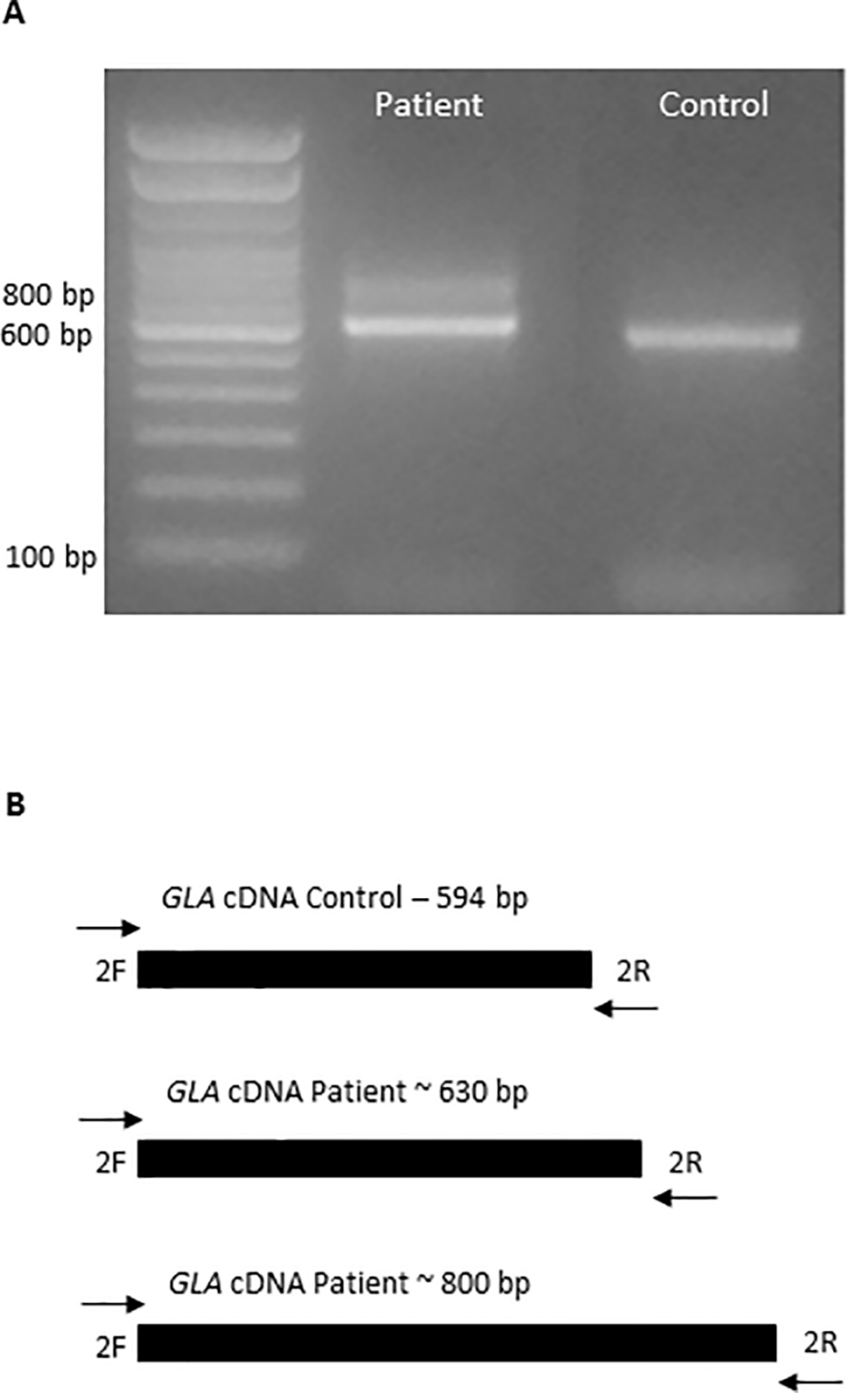
Aberrant mRNA transcripts formation caused by the insertion in the *GLA* gene. **(A)** Amplification of a cDNA fragment from patient and healthy volunteer by PCR, with primers cDNA 2F and cDNA 2R, showing formation of two fragments in the patient. **(B)** The diagram described the wild-type allele from healthy volunteer and the two aberrant alleles formed in the patient.

In order to determine the impact of the novel mutation c.801+2_801+3insT in the mRNA and protein, cDNA was sequenced and the defect was confirmed. The electrophoresis showed two transcript fragments produced from the mutated *GLA*, presenting approximately 630 and 800 bp, instead of the 594 bp fragment corresponding to the normal allele, which was not observed in the patient (**Figure 2**).

The cDNA sequencing determined the nucleotide sequence of two aberrantly formed alleles. The first transcript has a 37-bp insertion, occurring between methionine 267, the last amino acid of exon 5, and leucine 268, the first amino acid of exon 6. Thirty-six bases of the inserted sequence correspond to the initial region of the wild-type intron 5 and a thymine base corresponding to the insertion found in the patient’s DNA. This extra sequence leads to the insertion of 12 amino acids in the protein and promotes a frame-shift, generating a premature stop codon at residue 311 (p.Leu268Valfs*43).

**Figure 3:**
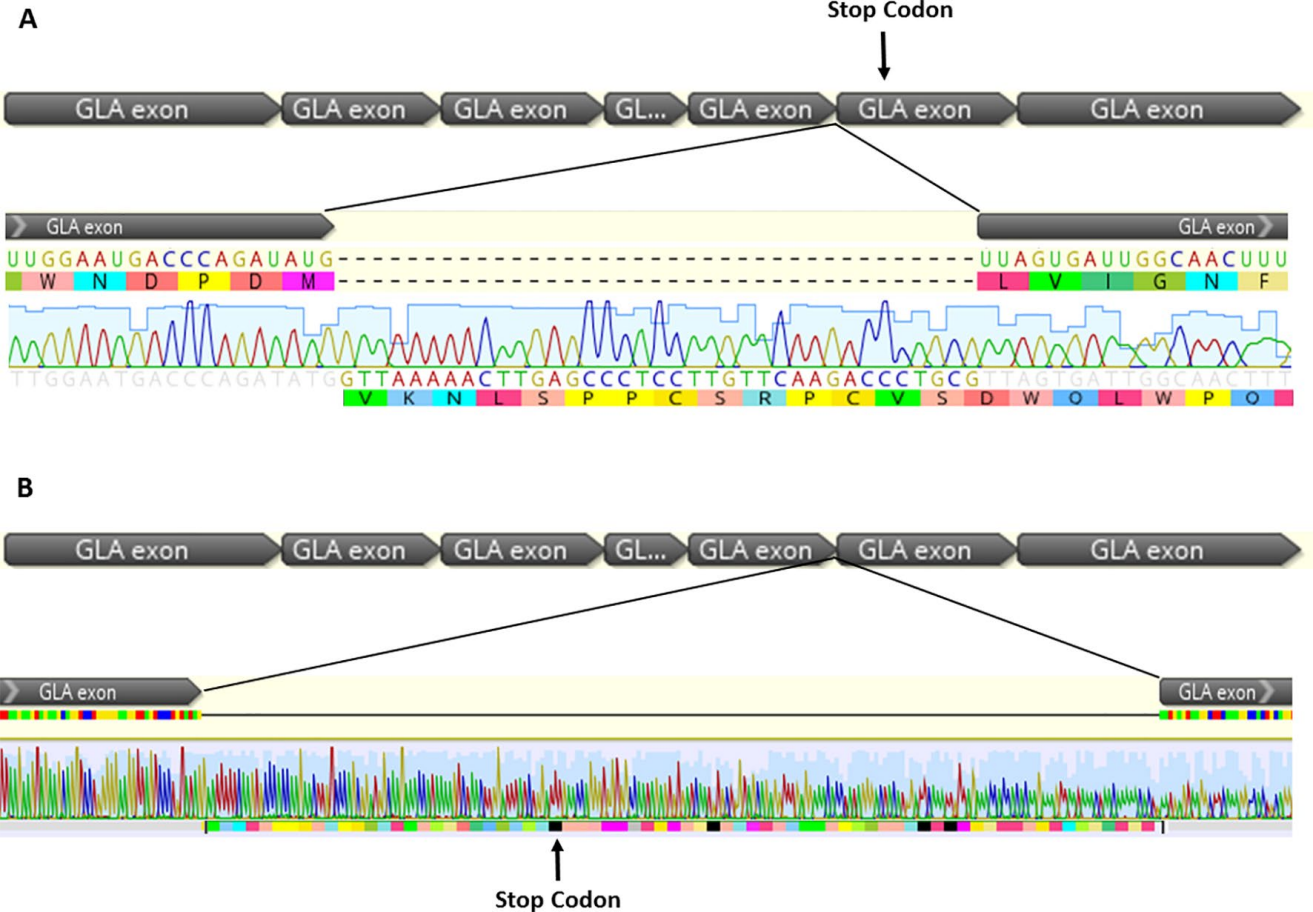
cDNA sequencing from two aberrant splicing events found in the patient, which occurs by the inclusion of intron fragments, forming cryptic exon into the mature transcript. **(A)** In the transcript 1, 37 bp were inserted corresponding to the first bases of the intron 5, plus the thymine insertion at position +3. The figure shows alteration of the open reading frame, promoting formation of a premature stop codon in the exon 6 (p.Leu268Valfs*43). **(B)** The 5’ splice site was not recognized in the transcript 2, and the excision of intron 5 was not observed. There was an insertion of 217 bp corresponding to intron 5 plus a thymine, corresponding to the base insertion at the genomic DNA, promoting a frame-shift and a premature stop codon in the intron 5.

In the second aberrant allele, the intron 5 was not removed and 218 bp was inserted in the wild-type sequence: 217 bp corresponding to intron 5 and a thymine corresponding to the inserted base in the genomic DNA. This insertion promotes a frame-shift and generates a premature stop codon at position 294 (p.Leu268Valfs*27). These results are shown in **Figure 3**.

## Discussion

FD occurs due to α-Gal A deficiency, an enzyme encoded by the *GLA* gene. *GLA* gene, located on the long arm of the X chromosome (Xq22.1), is organized into seven coding exons. The mRNA is formed by 1,290 bp encoding 429 residues with the first 31 amino acids being the signal peptide, which is removed to form the mature enzyme (Bishop et al., 1986; Garman and Garboczi, 2004).

Mutations in splice site regions can produce errors during the splicing, altering the RNA processing and leading to disease. The splicing process occurs by a specialized machine called spliceosome. This machinery is composed by ribonucleoprotein complex, which identifies specific regions of the pre-RNA, recognizing and promoting the intron’s excision and connection of exons (Grodecká et al., 2017; Abramowicz and Gos, 2018).

The *GLA* gene contains canonical exon/intron boundary sequences with GT (5’) and AG (3’) motifs at the end of the introns. Usually, classical splice site mutations affect the position +1 and +2 in the 5’ss and –1 and –2 position of the 3’ss (Krawczak et al., 2007). However, it is known that mutations in nucleotides close to the canonical splice site, as well as deep intronic variants, can cause errors in the splicing process, leading to disease (Ibrahim et al., 2007; Hori et al., 2013; Palhais et al., 2016). In the *GLA* gene, many polymorphisms located in non-coding regions are described. However, these polymorphisms do not cause splicing errors (Oliveira et al., 2008; Ferri et al., 2012). In the HGMD, only 14 intronic variants in the *GLA* gene located out of the canonical splice site are correlated with splicing errors. Among them, nine are located in regions near the canonical splice sites (positions +3 to +5); the other five are deep intronic variants.

Here we presented the case of a family with FD caused by a novel intronic variant: a thymine insertion in the position c.801+3 of the intron 5 (c.801+2_801+3insT), which disrupts the α-Gal A formation leading to the classical phenotype of FD. Three members of the family with FD symptoms present the variant in the *GLA* gene, being two brothers and a female cousin. All of them began to present FD symptoms as angiokeratomas, gastrointestinal alterations, acroparesthesia, and fatigue in the second decade of life. However, the suspicion of FD was only raised when the index case presented renal, neurological, and cardiovascular alterations, in the fourth decade of life. Just like the index case, the sibling has vital organs affected and α-Gal A reduced activity levels. However, the affected woman, despite presenting several symptoms of FD, still does not present alterations in vital organs probably due to random inactivation of the X chromosome. The variant found in this family results in two aberrant splicing events, which leads to the inclusion of intron fragments into the mature transcript (p.Leu268Valfs*43 and p.Leu268Valfs*27) and to the absence of the wild-type transcript.

In the transcript p.Leu268Valfs*43, the thymine insertion creates a novel donor splice site, 37 bases inside the intron 5. The novel donor splice site is recognized by the spliceosome, which promotes the excision of part of intron 5 and leads to intron inclusion. This intron inclusion causes an alteration in the open reading frame and promotes the generation of a premature stop codon and thus a nonfunctional protein. In the second aberrant transcript, the splice site was not recognized, and thus excision of intron 5 does not occur. Similarly to the generation of the first transcript, the open reading frame in this case was altered and a premature stop codon was formed.

Curiously, Li et al. (2019) recently showed the novel variant c.801+1G > A, present in the canonical splicing site of intron 5. They observed formation of two transcripts, the wild type and a transcript with 36-bp insertion. The authors demonstrated that in the aberrant transcript, the spliceosome recognized the nucleotide G at position 36 and GT at position 37 and 38 of the intron 5 as the 5’ss, thereby inserting the first 36 bases of intron 5 into the transcript. We observed the same event in the variant c.801+2_801+3insT, with inclusion of 37 bases in the first transcript, 36 referring to the intron 5 and a base referring to the insertion observed in the genomic DNA. However, in our study the wild-type transcript was not formed. Our results are in agreement with the findings of Shabbeer et al. (2006). They reported two single mutations at the donor splice site of the intron 5 in patients with classical FD (IVS5+3A > G and IVS5+4A > G). Similarly as demonstrated here with the variant c.801+2_801+3insT, the mutation IVS5+3A > G also resulted in two abnormal transcripts with intron inclusions and without the presence of the normal transcript.

Interestingly, variants in the c.801+3 position disrupt the recognition of 5’ss of intron 5 by the spliceosome, preventing the formation of the wild-type transcript leading to FD. It is suggested that in the 5’ss of the intron 5, the base adenine at position c.801+3 is a conserved base recognized by the elements of the spliceosome, and thus, variants in this position alter the interaction with the spliceosome machinery and therefore, the intron removal, forming aberrant transcripts.

Usually, as a way to prevent aberrant protein synthesis, the presence of the premature stop codon in the transcript leads to a process called nonsense mediated decay or RNA decay (Sterne-Weiler and Sanford 2014). This suggests that in the presence of the variant c.801+2_801+3insT, the protein is not formed, thus explaining the undetected α-Gal A activity in the index patient.

In summary, in the present study, we reported a novel *GLA* mutation found in a 46-year-old male and his family, diagnosed as having FD by clinical signs and undetectable α-Gal A activity. Our study showed that in the *GLA* gene a single insertion at the c.801+3 position of the 5’ss of the intron 5, despite not being in the canonical splicing site, caused the formation of two aberrant transcripts containing premature stop codons, leading to FD.

## Data Availability

The datasets for this manuscript are not publicly available because the data are under the care of the corresponding author and may be made available when necessary. Requests to access the datasets should be directed to João Bosco Pesquero, jbpesquero@gmail.com.

## Ethics Statement

The Research Ethics Committee of the Federal University of São Paulo, Brazil, approved this protocol (0585/07 and 0354/18). All procedures were followed in accordance with the ethical standards of the responsible committee on human experimentation (institutional and national) and with the Helsinki Declaration of 1975, as revised in 2013. All subjects involved in this study were older than 16 years at the time of the study (index case—47 years/Brother—37 years/Cousin—48 years). All three patients gave *written informed consent* agreeing to participate in the study and for the publication of their clinical cases. The consent was in accordance with the Declaration of Helsinki.

## Author Contributions

PV: conceptualized and designed the study, designed the data collection experiments, drafted the initial manuscript, carried out the analyses, reviewed and revised the manuscript, and approved the final manuscript as submitted.

MM: providing the samples analyzed in this work, analyzed the clinical symptoms of patients, reviewed and revised the manuscript, and approved the final manuscript as submitted.

JP: conceptualized and designed the study, drafted the initial manuscript, carried out the initial analyses, reviewed and revised the manuscript and approved the final manuscript as submitted. Corresponding author and *guarantor* for the article.

## Funding

This work was supported by grants from Fundação de Amparo à Pesquisa do Estado de São Paulo (FAPESP 2014/27198-8) and Coordenação de Aperfeiçoamento de Pessoal de Nível Superior —Brasil (CAPES)—Finance Code 001.

## Conflict of Interest Statement

The authors declare that the research was conducted in the absence of any commercial or financial relationships that could be construed as a potential conflict of interest.
